# The Drinking Water Tool: A Community-Driven Data Visualization Tool for Policy Implementation

**DOI:** 10.3390/ijerph19031419

**Published:** 2022-01-27

**Authors:** Clare Pace, Amanda Fencl, Lauren Baehner, Heather Lukacs, Lara J. Cushing, Rachel Morello-Frosch

**Affiliations:** 1 Department of Environmental Science Policy and Management, University of California, Berkeley, CA 94720, USA; baehnerl@berkeley.edu (L.B.); rmf@berkeley.edu (R.M.-F.); 2 Department of Geography, Texas A&M University, College Station, TX 69016, USA; alfencl@ucdavis.edu; 3 Community Water Center, Watsonville, CA 95076, USA; heather.lukacs@communitywatercenter.org; 4 UCLA Fielding School of Public Health, University of California, Los Angeles, CA 90095, USA; lcushing@g.ucla.edu; 5 Department of Public Health, University of California, Berkeley, CA 94720, USA

**Keywords:** Human Right to Water, environmental justice, water quality, drought, community-based participatory research (CBPR), cumulative exposure, domestic wells, water security, groundwater, European Science Shop

## Abstract

The Drinking Water Tool (DWT) is a community-driven online tool that provides diverse users with information about drinking water sources and threats to drinking water quality and access due to drought. Development of the DWT was guided by the Community Water Center (CWC) as part of the Water Equity Science Shop (WESS), a research partnership integrating elements of community-based participatory research and the European Science Shop model. The WESS engages in scientific projects that inform policy change, advance water justice, and reduce cumulative exposure and disproportionate health burdens among impacted communities in California. WESS researchers conducted qualitative analysis of 15 stakeholder interviews regarding the DWT, including iterative feedback and the stakeholder consultation process as well as stakeholder perceptions of the tool’s impact on California water policy, organizing, and research. Results indicate that the DWT and the stakeholder engagement process which developed it were effective in influencing policy priorities and in promoting interagency coordination at multiple levels to address water equity challenges and their disproportionate burdens, particularly among rural and low socioeconomic status areas and communities of color.

## 1. Introduction

Unsustainable groundwater withdrawals coupled with ongoing drought conditions in California threaten long-term access to groundwater for drinking and agriculture [1,2]. California’s 2012–2016 drought resulted in a declared state of emergency in 2014 [3] and an estimated 2600 domestic wells were reported to have run dry [2,4], with well failures reported 1.5 times more frequently in disadvantaged communities (disadvantaged communities are defined in the Safe Drinking Water Act and Water Code Section 79.505.5 as communities with a median household income (MHI) below 80% of the statewide MHI) [5]. California is again experiencing a statewide drought which is expected to worsen in coming years due to climate change; state agencies received more than 950 reports of dry domestic wells between 1 January and 12 November 2021 [6].

In addition to supply concerns, contamination from past and present industrial and agricultural practices threatens groundwater quality in California. Approximately 10% of California’s public water systems are currently out of compliance with federal regulatory standards for water quality [7]. Rural communities served by small water systems with fewer than 200 service connections experience a greater proportion of federal drinking water quality violations compared to medium and larger systems [8], as small systems face disproportionate challenges in meeting regulatory Maximum Contaminant Levels (MCL) due to a lack of financial, technical and economic resources to treat contaminated water or acquire new drinking water sources [9,10,11,12,13]. Communities served by water systems with elevated contaminant concentrations are disproportionately socioeconomically disadvantaged communities. Many of these are disproportionately Latinx, raising environmental justice concerns [11,12,14]. Additionally, communities served by domestic wells often face significant water quality challenges compared to those served by community water systems (CWSs). It is estimated that 1.3 million Californians rely on private domestic wells [15], which commonly serve rural, agricultural, and socioeconomically disadvantaged communities [16]. A recent statewide environmental justice study in California found that mean arsenic, nitrate and hexavalent chromium levels exceeded their respective MCLs for a greater proportion of people who used a domestic well compared to those who used a community water system, and that poor water quality in domestic well areas disproportionately impacted Latinx and communities of color [15]. As these contaminants pose cancer and non-cancer health risks [17], such groundwater contamination threats can contribute to cumulative exposures from multiple chemical stressors and disproportionate health burdens among the impacted communities [11,12,14].

To address chronic water quality concerns, in 2012 California passed Assembly Bill 685 [18], known as the Human Right to Water law, which recognizes the legal right to clean affordable drinking water for all Californians including disadvantaged communities and those served by domestic wells. Given the high level of reliance on groundwater by households in the state, the Human Right to Water law was strengthened by passage of the Sustainable Groundwater Management Acts (SGMA) in 2014, which established rules and processes by which sustainable groundwater pumping and management can be achieved by 2040. The SGMAs require the creation of new local Groundwater Sustainability Agencies (GSAs) responsible for developing Groundwater Sustainability Plans (GSPs) to ensure that groundwater protection strategies and usage achieve sustainability by 2040. Several state-owned data tools were developed to track progress toward achieving the goals established under the Human Right to Water and SGMA laws, including two key resources created shortly after their passage: the State Water Resource Control Board’s Human Right to Water portal [19], which tracks system compliance with regulatory standards, and the Department of Water Resources’ (DWR) SGMA Data Viewer [20], which makes datasets available to support GSP development and implementation.

Most state agency tools did not exist at the time the Drinking Water Tool (DWT) was launched in early 2020, and very few included all types of household water users. For example, in 2021 California Environmental Protection Agency’s (Cal-EPA’s) Office of Environmental Health Hazard Assessment (OEHHA) developed and released a framework to track progress toward achieving the Human Right to Water [21] for CWSs only, not domestic well communities. Meanwhile, the State Board has developed tools focused on domestic wells: the 2021 Aquifer Risk Map [22] and the GAMA program’s Domestic Well Water Quality Tool [23], both released in 2021 as part of the 2021 Drinking Water Needs Assessment [24] do not include CWSs. Presently, these State Board tools consider ambient groundwater quality from monitoring wells, not treated water delivered by community water systems, and give an incomplete picture of water quality differences in domestic well areas compared to those served by CWSs [15]. A final tool under development during the same period is the DWR’s Drought and Water Shortage Risk Explorer, which contains data layers related to drought risk and is designed to help smaller water systems and self-supplied communities (e.g., communities served by domestic wells) to better understand their potential drought vulnerability [25]. Currently, this tool contains only data on drought and social vulnerability, not drinking water quality. The distinctive features and novelty of the DWT in comparison to other similar tools include the fact that it was developed in a stakeholder-engaged process that was led by a community-based organization (the Community Water Center (CWC)) rather than a government agency, and that it integrates information related to (1) multiple drinking water sources (community water systems as well as domestic wells), (2) water quality as well as quantity (drought) information, and (3) socioeconomic information. We reflect here on the initial vision, creation, and impact of the DWT through a series of stakeholder interviews in an effort to better understand how community-driven data tools such as the DWT can catalyze solutions and promote progress towards water justice goals. Based on an analysis of interview themes, we demonstrate that the stakeholder-engaged process used to develop the DWT influenced discussion and decisions regarding the mechanisms by which to achieve the aims of California’s Human Right to Water law and protect vulnerable groundwater users in GSPs (under AB 685 and SGMA, respectively).

In the future, the DWT can continue to be leveraged by advocates and researchers to inform multi-level regulatory and policy deliberations to address water equity challenges and their disproportionate burdens, particularly among rural and low-income areas and communities of color.

## 2. The Drinking Water Tool

### 2.1. Drinking Water Tool Origin

In the absence of a comprehensive and accessible statewide tool, the Community Water Center (CWC), as part of the Water Equity Science Shop (WESS), sought to create an online Drinking Water Tool (DWT) [26] in 2017. The DWT offers an online platform that visualizes multiple water user types (CWSs and domestic wells) and multiple drinking water challenges (drought threats, water quality, access to treated water, and socioeconomic vulnerability) [26]. California residents were identified as one of the primary audiences envisioned as potential tool users, particularly those that rely on domestic wells and those on CWSs who want to learn about who manages their drinking water, whether there are potential issues with drinking water quality, and how to get involved in local water management. Organizations such as the CWC provide this information to community residents in areas where domestic well testing programs are available in the Central Coast and Central Valley of California, help to connect residents to domestic well water quality testing programs, and provide individualized, meaningful, and linguistically accessible results. Statewide, however, water quality monitoring and science communication remain sparse. Community organizations seeking to test and characterize drinking water quality among vulnerable communities reliant upon domestic wells and small systems require better tools to generate actionable information for their community residents.

Since 2004, the CWC has engaged communities and marshalled scientific resources to inform grassroots organizing and advocacy to advance water justice among some of California’s most disadvantaged communities. WESS is a research partnership between the CWC, the University of California, Berkeley and Los Angeles, and Cal EPA’s OEHHA. As a community–academic research collaborative, WESS draws upon the best practices of community based participatory research (CBPR) [27,28] and European Science Shop models [29] to advance sustainable and socially just strategies to improve drinking water quality. Key to CBPR is that study questions originate in the community and improve the lives of the people involved through the co-generation of knowledge and shared leadership throughout the research process [30], capacity-building, and environmental problem-solving [29]. CBPR benefits the scientific enterprise by improving its methodological rigor, public relevance, and policy reach [31]. Similarly, the concept of Science Shops posits that research is enhanced when communities are engaged in the generation of new knowledge as active participants rather than “receptacles” for “expert” knowledge [32]. Science Shops typically coordinate and execute participatory research, bringing together university researchers and community organizations to facilitate scientific research that responds to the needs and interests of all stakeholders [33]. WESS’s major innovation is its integration of CBPR and Science Shop models to catalyze community-driven research that improves drinking water policy and regulation, informs interventions, and advances environmental justice goals. Fundamental to the success of this approach is the strong foundation of effective working relationships and trust among WESS community and academic investigators, and the capacity to convene diverse stakeholders in conversation.

The CWC was uniquely suited to lead the development and guide the design of the DWT through a strategy that leveraged the scientific expertise of the WESS and engaged diverse stakeholders throughout the DWT development process. To inform and launch the DWT, the CWC established an advisory committee and a process to determine stakeholder needs related to achieving universal access to clean affordable drinking water in California. This convening of community, academic, and agency stakeholders shared ideas related to advancing California’s Human Right to Water and equitable SGMA implementation. Participants included members of the La Asociación de Gente Unida por el Agua (AGUA) Coalition, Environmental Justice Coalition for Water, the Bay Area Environmental Justice Networking Group, agency staff and board members from the State Board, the California Department of Water Resources (DWR) and the California Department of Public Health (CDPH), as well as academic researchers from UC Davis’s Center for Community Change, UCLA Luskin Center, UC Merced, and UC Santa Cruz, among others. Through these conversations, local knowledge, and recent experiences navigating the 2012–2016 drought emergency, the CWC identified a critical data gap: unregulated water sources, including domestic wells and small water systems with fewer than five service connections.

Not only are these sources not routinely monitored (Monterey County, a notable exception, monitors local small water systems serving 2–4 connections) or regulated for water quality; many of them have experienced drought-related well failures and in general, little data exists to locate communities reliant on these sources. Therefore, although California has a Human Right to Water law (AB 685) that legally recognizes the right to clean, affordable drinking water among all residents, including those served by domestic wells, this data gap raises significant challenges for informed regulatory and intervention strategies that address the drinking water problems faced by these small and largely rural communities. In particular, GSAs have only considered water quality to a limited extent, if at all, for domestic well users in the development of GSPs, and reviews of GSPs warn about domestic and public well failures if plans are implemented as written [34,35]. A common push back from GSAs is that they lack easily accessible information to reliably assess the impact of their plans on domestic well communities.

**Figure 1:**
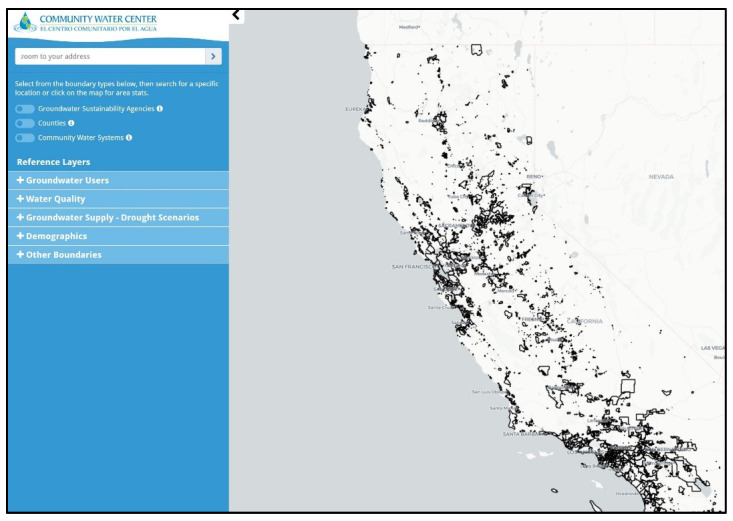
Screenshot of the Drinking Water Tool displaying community water systems for the entire state of California (https://drinkingwatertool.communitywatercenter.org/ca-water/, accessed on 30 November 2021).

The DWT [26] (Figure 1) was designed to fill these critical data gaps and provide an interactive, English/Spanish-bilingual online web tool to meet the needs of community and agency stakeholders. The process of developing the DWT was iterative; WESS researchers first developed methods for integrating multiple statewide data sets to begin identifying areas primarily dependent on domestic wells and estimating the groundwater quality of domestic well areas. Existing secondary data sources used and modified included the Tracking California’s Drinking Water Systems Geographic Reporting Tool, used for locating Community Water System Boundaries [36]; the Department of Water Resources’ Online System for Well Completion Reports [37], used for locating domestic wells; demographic data from the 2010 Census [38], the 2013–2017 American Community Survey [39], and the State Board’s Water Quality Monitoring Database [40], used for estimating CWS water quality; and the State Board’s Groundwater Ambient Monitoring and Assessment (GAMA) program [41], which includes sampling information collected from a subset of domestic, monitoring, and public supply wells.

The WESS research team developed draft versions of each layer that were then shared and refined in conversation with the CWC. The CWC contracted additional expertise, including an applied geographer who co-managed the DWT project (co-author Fencl), a hydrogeologist who led the development of data layers related to drought threats, and GreenInfo Network, a non-profit specializing in spatial webtool development, to create the DWT web interface. Once a draft tool was developed that met internal goals, the CWC hosted two large workshops attended by approximately 85 diverse stakeholders. Feedback from those meetings was incorporated into further refinements of the DWT data layers and website interface. This process of iterative feedback and collective decision-making was critical in developing a final product that had support from state agencies and local stakeholders in order to enhance the dissemination and use of the final product.

In sum, the DWT is unique among many online environmental spatial data tools in that its creation was guided by a community-based organization (CWC) and is the product of a community–academic collaboration through the WESS, in contrast to many of the state agency-led tools described previously. Second, the DWT development process was iterative and relied heavily on feedback from a diverse stakeholder advisory group. Third, the final DWT contains two unique portals, each designed to serve a distinct set of stakeholders, namely, community members and policy makers.

### 2.2. Drinking Water Tool Overview

**Figure 2:**
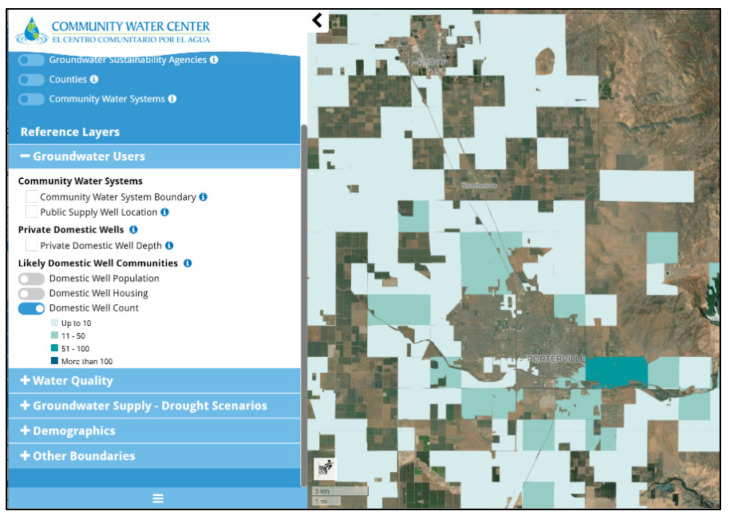
Screenshot of the Drinking Water Tool showing the Groundwater Users reference layer with the Likely Domestic Well Communities option set to Domestic Well Count for a particular region.

The DWT provides interactive statewide information about GSAs, counties, and CWSs. A set of Reference Layers (see list in Figure 1) includes information for CWSs and domestic well users (Groundwater Users) regarding the source and estimated quality of their drinking water (Water Quality) and drought risk estimates for groundwater users in the Central Valley (Drought Scenarios). Specifically, the DWT highlights those communities across the state that might be vulnerable to groundwater challenges such as poor groundwater quality and drought risks that could adversely affect their long-term access to safe and affordable drinking water. Structurally, the DWT contains more than 40 state-wide spatial data layers that users can visualize (Table A1), and while many of the data layers are repurposed from existing state or federal datasets with limited manipulation, there are two novel research products developed specifically for the DWT. WESS researchers contributed the first novel layer, which provides the first statewide spatial characterization of areas that self-supply from domestic wells [42] (shown in Figure 2) as distinct from the communities served by CWSs [36]. The second novel layer is the Central Valley well impact analysis, a set of drought scenarios for domestic and municipal supply wells in the San Joaquin Valley, which displays information on potential drought vulnerability [43]. This layer represents one research project and is displayed as eight different layers in the DWT, based on a combination of drought scenarios and impacted wells. It considers how declining groundwater elevations under a set of possible drought conditions might reduce well production potential by predicting the likelihood of increased pumping lift, well screen clogging, and wells running dry [44], and provides DWT users with an estimate of the number of impacted domestic and public supply wells and the associated costs of a future drought equivalent to that experienced in California between 2012 and 2016 [43].

Additional layers communicate water quality estimates for arsenic, nitrate, hexavalent chromium, and 1,2,3-trichloropropane (1,2,3-TCP) representing the nine-year average delivered water contaminant concentration for each community water system and nine-year average groundwater quality that may affect domestic wells; the data used to construct nine-year average water quality were compiled by OEHHA for CalEnviroScreen 3.0 (CES 3.0) [45]. The resulting layers differ in that the DWT displays water quality at a smaller spatial resolution (CWS and 1 × 1 mile grid square), whereas CES 3.0 water quality is aggregated to the scale of Census Tracts [45]. The DWT includes boundaries and characteristics for alluvial groundwater basins, political jurisdictions, and demographic data from the 2017 American Community Survey (ACS) [39] at various census geographies for median household income (MHI), disadvantaged community (DAC) status (based on MHI), and race/ethnicity. The DWT’s data layers can be visualized using a web browser, and users can download most of the datasets for their own analysis.

**Figure 3:**
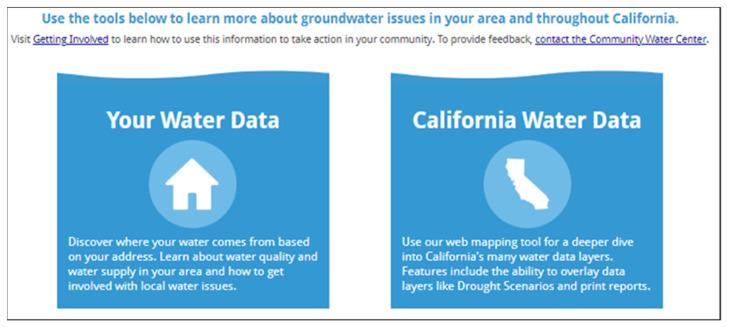
The Drinking Water Tool landing page displaying the two portals: Your Water Data and California Water Data.

A critical element of the DWT is its capacity to provide community users with information regarding where their water comes from, who manages their water supply, nine-year averages of groundwater quality in the area where they live, potential threats to their groundwater supply from future droughts, and how to get involved in local groundwater management decisions. The community-focused component is one aspect of the two portal design of the DWT (Figure 3): a “Your Water Data” link contains resources developed for community members, whereas the “California Water Data” portal provides data visualization of the available spatial layers (Table A1) for policy makers, researchers, and advocacy groups.

### 2.3. Drinking Water Tool Public Release

The DWT was released in February 2020 at a widely publicized webinar with over 220 attendees including representatives from government agencies, non-profit groups, academic institutions, and community members. In its first year, a median of 206 visitors used the site each month, and there have been over 3100 site visitors as of 21 January 2021. DWT data has been downloaded and used for a variety of projects and initiatives related to the implementation of California’s Human Right to Water and SGMA laws. Following the launch of the DWT, the CWC and WESS researchers have participated in meetings on the implementation of GSPs and conducted technical workshops with regulatory scientists, other university researchers, and several federal and California state water agencies. These meetings and workshops provided a forum to coordinate research activities and share data and methods to support the Human Right to Water by identifying domestic well communities and estimating their ground water quality. Through these efforts, the WESS sought to promote community–academic collaboration and synergize efforts between community groups, researchers, and regulatory scientists in order to inform policy and regulatory efforts to address drinking water disparities.

## 3. Materials and Methods

WESS researchers recruited interviewees for semi-structured interviews using a stratified approach that identified categories of stakeholders (government agency, academic, and non-profit) and conducted interviews with fifteen stakeholders during late summer of 2021, about 1.5 years after the DWT’s public launch. All interviewed stakeholders had significant expertise and a connection to the DWT development process, drinking water data analysis, or statewide policy implementation.

Key informants were identified and recruited based on one the following criteria: current or former affiliation with the DWT development team, attendance at one of the stakeholder webinar consultations, or subject matter expertise (i.e., a state agency employee working on similar tools or research areas or with decision-making roles within relevant agencies). Interviews ranged from 40–60 min and took the form of semi-structured conversations with individuals or small groups, with transcripts generated by Zoom. Several of the authors analyzed and coded de-identified transcripts using Dedoose software (SocioCultural Research Consultants, Los Angeles, CA, USA). Analysis relied on a mix of inductive and deductive coding; the authors of this paper used interview questions to develop a first round of codes, tested and refined the codes on a subset of the interviews, and constructed a codebook (Table A2). Two researchers applied the codebook to the entire set of interviews, then the authors conducted theme analysis on excerpts grouped by the final codes.

Major themes from the interviews revolved around the following topics: (1) stakeholder engagement, (2) impact of the DWT on state agency decision-making, and (3) perceptions of the DWT as a data source developed by a community-based group (Table 1). Results are presented for each of these themes in the next section, with coded excerpts grouped by the interviewee’s organizational affiliation (academic, non-profit, or government agency). In addition to many of the authors’ own experiences as part of the DWT development team, the data analyzed in this paper represent the views of seven non-profit employees, five government employees, and three academic researchers. The affiliation of several interviewees changed between the time the DWT development began and the time of stakeholder interviews. For this reason, all participants were categorized by their professional role at the time of the stakeholder interview. To better clarify opinions of different and sometimes overlapping categories of informants, we define “DWT developers” as WESS researchers, DWT consultants, and current CWC employees interviewed for this paper. Opinions attributed to “non-profit informants” refer to non-profit groups, community organizers and advocacy groups, excluding the CWC.

## 4. Results

### 4.1. A Collaborative Process Is Equally as Important as the Final Product

The DWT development process as steered by the CWC involved a unique collaboration between university researchers, the Cal-EPA’s OEHHA, technical consultants, and CWC staff. Multiple interviewees indicated that the relationships among these different collaborative elements were critical to ensuring the success of the project and its implementation as well as to data acquisition, receiving timely and expert feedback on analysis and interpretation, and to the messages delivered on the online tool. In reflecting on the role of WESS researchers as collaborators, an agency informant and previous collaborator on the DWT development team stated, “[WESS researchers] played a really big role in helping translate between technical [components] in our project [and the final tool].” Similarly, the WESS research group acknowledged that the DWT collaboration would not have been possible without the local knowledge, expertise, and community engagement that the CWC brought to the partnership. Multiple stakeholders communicated that there was a benefit in having the DWT developed by a community–academic collaboration. The CWC’s role as an environmental justice advocacy group improved the perceived relevance of the DWT to the communities involved, and engendered trust among other non-profit groups. External stakeholder engagement further improved the data, methods, design, and accessibility of the DWT. Early in the tool development process, stakeholders shared knowledge and ideas about the best publicly available statewide datasets. WESS researchers refined their methods based on stakeholder feedback in which they compared their water quality results with those of GAMA’s Domestic Well Water Quality Tool (the State Water Board GAMA Program, in coordination with the U.S. Geological Survey, have tools focused on groundwater quality for domestic wells and very small systems that were developed as part of the 2021 California Drinking Water Needs Assessment program [24]). This served as an additional level of data quality control and led to further modification and improvement of the WESS researchers’ water quality model. Ongoing internal feedback from CWC staff not directly involved in the DWT development team helped to inform the choice to develop a two-portal model and the decision to tailor the DWT to different audiences through the two portals, and aided in refining the language and the Spanish translation used in the community-facing “Your Water” portal.

The interviewed DWT development team members noted that “the stakeholder advisory group was really important in the process […] we had big meetings where we solicited input as to what [stakeholders] thought should be in [the DWT].” In the early planning stages, the development team articulated their vision for the DWT as a product dynamic enough to evolve based on progress in data availability, emerging water issues, and evolving community identified concerns. DWT developers explained, “We envisioned the tool at different stages,” and relied heavily on the stakeholders to “consider a range of possibilities.”

There were unanticipated co-benefits of increased collaboration and conversation around water equity issues: the stakeholder webinars presenting the DWT development project updates served as a forum for state agencies and others who were doing related yet independent and parallel work to come together and generate ways to increase inter-agency collaboration. According to interviewees across agency, academic, and community-based organizations, the external stakeholder consultation process during development of the DWT was equally as important as the final product. A non-profit interviewee commented, “A large part of the power of [the DWT] was the conversation that developed through the stakeholder engagement process,” further elaborating that “community engagement gets folks talking and advances the conversation on water justice.” Another advantage of community-engaged stakeholder-led research is that it “improves communication between different government agencies and between non-profit and government entities.” This can lead to additional conversation and collaboration that is ultimately critical to achieving water justice, as it brings decision makers and policy-making agencies together with those who are impacted most by the policies and decisions in question.

All interviewees agreed that the transparency engendered via the stakeholder consultation process was critical for improving the methodological rigor of the DWT. Reflecting on the stakeholder engagement process, a DWT developer explained that the presence of the external stakeholders “was helpful because it was very transparent, we had regular webinars, and we were pretty clear about what we were doing and how we were using the data.” Another theme that emerged in interviews was the value of inter-agency communication in advancing the conversation on water justice. Agency interviewees agreed that the conversations they participated in as stakeholders in the DWT development process were valuable to their work in terms of how they approached the issue of incorporating domestic wells, especially because, as one agency interviewee recalled, “at the time, so many state agencies were not talking to each other” and the advisory committee meetings for the DWT became “a platform for that communication.” According to a non-profit interviewee, “the WESS played an important role in bringing the domestic well issue into the implementation space,” and “brought people together [to have conversations] in a way that wasn’t happening at the time”. Another government stakeholder felt that “the interaction was successful because we were sharing thoughts and ideas and approaches.”

According to a member of the DWT development team, “when I reflect on our work during [the DWT planning phase], I actually think that those meetings to coordinate what everybody was doing on domestic wells, talking about things like… where is the data? How do we interpret it? I think that was a really valuable piece of [the WESS’s] work.” Deeper collaboration and conversation around domestic well-related water equity issues, particularly around the unique data and policy gaps, was a critical contribution to the process of implementing California’s Human Right to Water and SGMA laws.

### 4.2. Putting Domestic Wells on the Map

From the perspective of DWT developers, the DWT Central Valley well impact analysis has been influential in efforts to achieve the Human Right to Water for domestic well communities and the SGMA’s groundwater sustainability goals. It laid the groundwork for generating new data that is informing both activity around drinking water quality and access and SGMA implementation and drought-planning efforts. For example, under SGMA guidelines GSAs representing the twenty critically overdrafted groundwater basins (high and medium priority basins) were required to submit their initial GSPs in January 2020 to the DWR, followed by a period of public review and comment. According to the SGMA, GSAs are responsible for setting their own management criteria; however, an interviewee explained that “in the event that a plan is determined to be inadequate by the Department of Water Resources, then that groundwater sustainability agency has [several] months to revise the plan. If they don’t [adequately] revise the plan, the Department of Water Resources and State Water Resources Control Board will come in, deem the GSP to be inadequate, and the State Water Board will intervene.”

Thus, the public comment period represents a critical window for review and refinement of GSPs and an opportunity to evaluate a plan’s environmental justice implications. While state agencies may, according to a state agency interviewee, be “relying on source data rather than these tools,” NGOs and others are able to use tools like the DWT to inform public comments. Critiques of GSPs that emerged during the comment periods expressed concern about how groundwater management criteria are being set, “particularly with respect to who has representation in the decision-making process, and how the water needs of an irrigation district are balanced against the water needs of communities served by domestic wells,” per a DWT developer.

In an extension of the Central Valley well impact data layer [43] developed for the DWT, the tool’s data were used to estimate the number of people threatened by SGMA implementation in an analysis of submitted GSPs [46]. This report, funded by the Water Foundation, elevated the fact that many plans for groundwater sustainability failed to consider domestic wells [46], while further studies confirmed the vulnerability of domestic wells [34] and public supply wells under the SGMA [35]. A DWT developer described the Water Foundation’s extension of the Central Valley well impact analysis in the DWT as “the first time someone brought to the attention of the regulatory agencies that they were clearly setting management criteria based on the priorities of the people who were sitting around the table. Which was farmers, which puts domestic well owners at a serious disadvantage” because domestic wells tend to be shallower and run dry before deeper agricultural wells in the surrounding basin.

By leveraging data made available by the DWT, environmental justice groups advanced the conversation on water justice by elevating inequities in water justice among disadvantaged communities. Furthermore, the CWC used the tool’s Central Valley well impact data layer [43] in their critique of the Kings River East GSP, leading to the inclusion of domestic well communities in drought planning efforts in the Kings River plan. Data layers in the tool continue to guide the CWC’s ongoing technical assistance projects, outreach, and project implementation.

As a next step, a CWC staff member interviewed as part of this research discussed plans for using the results of the DWT Central Valley well impact layer and related drought analysis for ramping up their advocacy about drought preparedness and advocating to have an interagency drought preparedness plan between state agencies. For example, CWC staff noted that this is particularly important because “SGMA is not totally online yet, and the most vulnerable communities don’t have [drought] plans”. They feel that the most efficient means to address this would be a collaborative effort that draws on the resources and expertise of multiple water agencies and other diverse stakeholders.

### 4.3. Deliberations on Tool (Dis)trust

Conceptually, the DWT integrates and analyzes secondary data from state agencies in order to enhance community involvement in issues of water justice, providing an avenue for community-based organizations such as the CWC and others to engage with policy and regulatory decision-makers. Existing relationships and trust between those in the water justice space likely contributed to uptake by non-state organizations. Convincing state agencies to adopt certain layers developed for the tool was more variable; certain agencies adopted the data layers, while others were more reluctant. Interviewees reflected on the hesitation by state agencies to use DWT data layers. First, differences arose among those we interviewed about their views on the DWT as a tool developed by a non-profit, environmental justice organization with clear advocacy goals. Researchers and non-profit interviewees expressed trust and confidence in the tool. Second, views on the utility of the DWT differed based on the scale of the research question and policy objective of specific legislative mandates. Third, timing and availability played an important role; the DWT was released after the deadline had passed for GSA priority basins to submit their initial plans. Despite this, interviewees affirmed the importance of the DWT in pushing policy discussions further by literally putting domestic well communities on the map, and provided an independent analysis against which state agencies could compare and externally validate tools they were developing internally.

In general, academic, and non-profit interviewees felt comfortable using DWT data and cited evidence of incorporating this information into their own research, advocacy, and planning activities. A clear contribution of the DWT, as identified by a non-profit interviewee, was the work WESS researchers did to “clean up the OSWCR [Online System for Well Completion Reports (WCRs)] data and make it useful”. The OSWCR database is maintained by the Department of Water Resources and contains the construction date, depth and approximate location of wells drilled in California after 1910. By extracting wells by use type (domestic) and combining it with other secondary data sources from other agencies, WESS researchers improved the resolution and more accurately defined the boundaries of the communities served by domestic wells [42]. The DWT’s domestic well areas layer (Figure 2) was the first statewide attempt to improve the utility of the fully digitized OSWCR dataset to approximate populations served by domestic wells by translating counts and locations of wells into an estimate of the populations reliant on those wells while making it more easily accessible to the public and non-technical users. (One previous study by Johnson, T.D. and Belitz, K., (2015) relied on a subset of non-digitized well completion reports—635,000 scanned WCRs of an estimated 1–2 million total WCRs) [47]. As one academic interviewee explained, prior to the DWT “there [wasn’t] enough fine resolution data on domestic well users in particular” citing the need for further refinement in order to achieve the Human Right to Water in self-suppling communities. A state agency interviewee agreed that the DWT’s domestic well mapping was “the biggest contribution of the DWT… because [locating domestic wells] is the biggest gap that we have in the state even now.” Not only that, as one academic explained, in comparing the DWT to an existing state agency tool: “If I had to choose abstractly, I was more comfortable with the drinking water tool in terms of the validity, but what [name redacted] had was better than what the agency was using before” and explained that even though the DWT layers weren’t used by [name redacted], “it wasn’t an analytic concern”. Communities served by domestic wells are disproportionately rural and socioeconomically disadvantaged, making accurate mapping critical in efforts to ensure equitable implementation of California’s water laws.

In addition to addressing the analytical rigor of the DWT’s data layers, non-profit interviewees explained the importance of the CWC’s reputation and history of successful community engagement, which solidly anchored their identity as a community environmental justice advocate in the Central Valley. This local trust was critical to advocacy groups’ acceptance of the information in the DWT. Indeed, in several of these communities, non-profit interviewees have seen a strong “distrust of outsiders, of NGOs coming in, academics coming in with data that they’re not so sure that they can trust.” The CWC’s involvement in and leadership of the DWT development process was critical to the tool’s trustworthiness among the communities they serve, both residents and other non-profits. As one non-profit interviewee pointed out, “rural development moves at the speed of trust,” elaborating that “there is a lot of stuff that we really feel strongly needs to be communicated to the residents in the appropriate way and this required a high level of awareness in order to have an impact in a community.” This person continued by sharing that “It’s definitely been a challenge on how to present [drought and water quality issues] the correct way. So that [the data] will be accepted by the residents.” One of the goals of the DWT was to provide bilingual resources and a community-directed approach to help frame water challenges and solutions in a meaningful way, which interviewees affirmed was achieved.

The other side of the CWC’s reputation as an environmental justice leader in the state is that there was a mixed reception from different government agencies; while several readily adopted DWT layers, others expressed more reluctance. There are several possible explanations for this skepticism; for example, as one DWT developer and former CWC staff person suggests, perhaps “because of the relationship that CWC has in the [Central] Valley….when you have groundwater sustainability agencies that are primarily irrigation districts, there may be a hesitancy to use data [perceived to be] curated by an environmental justice group.” Another explanation for reluctance in uptake of the DWT data layers is limitations in the underlying data. Although DWT developers used the best statewide data available, a part of these data lacked the resolution and specificity of data collected internally by the CWC through well testing programs and other local efforts. For example, the Monterey County Health Department is one of the few counties in the state with an active Small Water System program that monitors systems with 5–14 service connections; the Department developed a map of the county’s small local water systems that displays water quality data for several contaminants based on actual samples rather than modeled data. Analytically, the water quality data in the DWT represent nine-year averages. While these capture three federal regulatory cycles established under the Safe Drinking Water Act (SDWA), they lack the specificity of local well monitoring programs. A non-profit group working on the ground to implement the SGMA commented that “what we’re trying to do isn’t so much on the research side, but on the practical inside…we’re actually working face-to-face with well owners and getting questions from them directly.” These interviewees felt that while the DWT was a step in the right direction, a “strategy to provide individualized results and data to domestic well users in the field would be enormously beneficial,” yet that is something currently outside the DWT’s capabilities.

One state agency interviewee was skeptical of the water quality data layer developed by WESS researchers specifically for the DWT, preferring to create similar data layers internally. This agency interviewee felt that “there’s a difference between us referring to a tool for external validation, or ideas for how we might be looking at GSPs or looking at a [proposed] intervention, there’s a difference between that and recommending use of a tool for a specific purpose, or relying on a tool for a decision.” They continue, stating that “I think [the DWT] has been really useful for us informally, in implementing SGMA, but it hasn’t necessarily risen to the level of formally blessing the tool or relying on the tool.” One possible reason for the push to create certain layers internally, regardless of the DWT’s parallel efforts, is that the research question and policy objective matter. As one state agency interviewee explained, “the CWC tool, from my view, the way you run into it is by investigating the Human Right to Water issue to begin with. You wouldn’t run into it if you were doing research on [water quality in a specific geographic] area. I don’t know if that line of research would lead you to the Drinking Water Tool.” Similarly, for GSAs, which may need additional details or ground truthing, the statewide scale of the DWT was too coarse, especially around groundwater quality.

Despite hesitancy by a few state agencies to adopt DWT data layers directly, the tool may have helped to accelerate related agency efforts to assess domestic well risk and drinking water quality. One state agency interviewee affirmed this scenario: “the drinking water tool definitely had an influence” on the level of attention on domestic wells. The DWT’s domestic well and water quality layers served as sources of comparison and external validation in the subsequent development of state tools. Though the DWT data layers were not used directly in state tools, the work of the WESS likely influenced the conversation around data availability and data use through state water agency involvement as stakeholders in the DWT development process. In the context of the SGMA, one of the CWC staff interviewed explained that the DWT is a valuable regional tool and can alert advocates and GSAs alike to “hotspots” for additional ground truthing, suggesting that additional data collection and monitoring would be needed to improve localized data and results dissemination strategies in the field. Interviewees agreed that the DWT’s first step of locating domestic well areas with publicly available statewide data remained “very important to move the conversation forward with regard to locating domestic wells, estimating drought risk, and making this data accessible to planners.”

The DWT’s water quality layers incentivized external validation of state tools. The State Board’s GAMA program worked with the WESS to compare the results of the GAMA program’s Domestic Well Water Quality Tool [23] and the DWT’s estimated water quality values. The GAMA program staff felt it provided a reality check that “the things they [GAMA] were doing with the data [were] representing the actual conditions out there.” An agency interviewee commented on the fact that the WESS and GAMA consulted on data sources and approaches early in the GAMA tool development process, then constructed similar yet independent projects which were later compared. “We ended up sort of going on parallel paths, and then comparing what we’ve done.” This was useful, because “when we’re out there and were making all these assumptions and estimations, it’s helpful to have someone else use a different approach and come up with a similar outcome. It’s useful for us and useful for others. The value is external–different groups are coming up with different tools and they can be compared.” The results of this internal assessment led to further refinement of the DWT’s water quality layers, catalyzed new analyses by the State Board to look at domestic wells and update community water system boundaries, and promoted data exchange.

In addition to the larger stakeholder meetings, WESS researchers and the CWC consulted directly with state agencies in the context of the tools the agencies were developing. With respect to the DWR’s Drought and Water Risk Score Explorer, agency developers participated in conversations and webinars with WESS researchers who sought input on locating domestic wells and assigning sociodemographic variables. Similarly, developers of GAMA’s Aquifer Risk Map and Domestic Well Water Quality Tool reached out to WESS researchers to discuss overlaps in data sources and methods. These conversations helped accelerate their own projects to assess domestic well risk and drinking water quality.

Following the release of the DWT, WESS researchers refined the domestic well areas layer to produce a higher-resolution map of populated areas served by domestic wells. The WESS researchers improved on the domestic well areas layer currently available in the DWT v. 1.0 for v. 2.0 using a process of dasymmetric mapping to disaggregate 2016 population estimates and assign population to the populated portions of census blocks based on residential parcels and building footprints [48]. Cal-EPA’s OEHHA integrated these refinements in population estimates into CalEnviroScreen (CES) 4.0, a screening tool for assessing cumulative impacts, environmental hazards, and chronic social stressors [49]. Notable, however, is that OEHHA is a co-founder and ongoing research collaborator with the WESS. In addition, as the scientific arm of Cal-EPA, OEHHA does not have direct regulatory authority over drinking water quality, SGMA implementation, or drought response.

## 5. Discussion

Both the DWT and the process and partnerships that created it had certain strengths and limitations. For example, one advantage of developing the DWT through an academic–community partnership is that it facilitated better acceptance among other non-profits, researchers, and state agencies (particularly those focused on water justice), as evidenced by interviewees who reported using the DWT data and methods in related projects. A downside was that other agencies did not utilize certain elements of the tool, including some GSAs and state agencies with their own policy/research goals that were reluctant to adopt a tool that they did not design. Another strength was the statewide nature of most of the DWT layers, and the fact that one of the novel layers created for the tool (the domestic well area layer) brought together diverse agency datasets, put domestic well areas on the map for the first time, and facilitated the CWC’s drought preparedness advocacy campaign for domestic users. However, one trade-off of taking this bird’s eye view was the lack of more granular information that users on the ground wanted and could have benefited from.

In addition, although the tool may not have been taken up and adopted by all state agencies in the way originally hoped for by the DWT development team, there was an unanticipated benefit of the stakeholder engagement process: the DWT catalyzed important conversations and collaboration and accelerated parallel efforts undertaken by agencies as they began creating their own tools to identify domestic wells with water quality and drought concerns. While the DWT alone did not necessarily bring about change, it was effective when leveraged by community groups in their advocacy (e.g., in the case of GSP comment letters) to ensure that vulnerable communities reliant on domestic wells were accounted for in policy implementation. Moreover, and by design, the inclusion of a set of broad stakeholders in building the DWT positively impacted the decision-making process by creating a forum and space to advance important conversations by providing accessible and actionable environmental justice analysis and building trust and relationships that facilitated and improved communication among agencies and across diverse stakeholders. By combining strong collaborative leadership, independent research utilizing publicly available statewide data, and the use of study results in community organizing and outreach, community-engaged efforts such as the DWT can influence inter-agency relationships and create opportunities for new conversations about critical knowledge gaps.

In thinking more broadly about how the work of the WESS contributes to strategies for long-term sustainable change, we reflect on past research demonstrating the value of collaborative governance in solving complex public health issues [50]. Similar to our stakeholder process, collaborative governance involves constructively engaging people in decision-making across the boundaries of public agencies, levels of government, and the public, private, and civic spheres [50]. Research on perceptions of trust and credibility demonstrate that science translates into environmental decision-making when it is perceived to be salient, credible, and legitimate [51]. Ultimately, the CBPR approach undertaken to develop the DWT as an environmental justice tool represents an important step toward actually achieving the Human Right to Water.

Simultaneously, we recognize that water quality and drought planning exist within a pre-established political context in which agricultural and industrial interests have historically had greater representation in decisions regarding water resource allocation than marginalized communities (i.e., domestic well users and disadvantaged communities). While the language of the Human Right to Water laws aims to change this by specifically naming disadvantaged communities, more work remains be done to collect and effectively disseminate critical data in ways that promote inclusion of marginalized groups in California’s drinking water policies.

In considering the future of the DWT, the developers expressed interest in improving the quality of the underlying data layers, including refinement of the domestic well areas layer, incorporation of more recent and accurate domestic well water quality testing data, and information that tracks and forecasts drought-related threats to drinking water access.

## 6. Conclusions

The WESS, a sustainable collaborative around water justice, is fostering interagency collaboration, prioritizing vulnerable domestic well communities, and setting the stage for improved coordination between research, government, and advocacy actors. Community-engaged strategies to develop the DWT helped to shape conversations among stakeholders and elevated the importance of environmental justice when implementing policies to ensure California residents’ access to clean and affordable drinking water.

## Figures and Tables

**Table 1:** Summary of main findings: pros and cons of the DWT development process and resulting data.

**Stakeholder engagement process** Pros Catalyzed conversations and accelerated parallel analytical efforts and data sharing Improved communication between agencies Improved rigor and relevance of the Drinking Water Tool (DWT) Led to deeper collaboration to address policy gaps Cons * Stakeholder engagement is time intensive (* this is not a finding, just a fact) Perceived utility of DWT depended on stakeholders’ affiliations and objectives
**DWT is a resource developed through a community engaged process and led by an environmental justice (EJ) group** Pros Improved relevance to community members Engendered trust among advocacy groups, researchers, and some agencies Cons Some agencies expressed hesitation to use certain data layers Utility of DWT depended on policy objective and scale of research question
**Novel data layers developed for DWT** Pros Highlighted and filled data gaps Incentivized external validation of state data tools Brought together diverse agency datasets Improved the utility of domestic well report dataset Cons Some state agencies preferred to develop their own versions of water quality tools using slightly different assumptions Statewide approach lacked granular information needed on the ground
**DWT impact on policy implementation** Pros DWT was leveraged by community groups in their Sustainable Groundwater Management and drought relief advocacy Influenced Groundwater Sustainability Plans (GSPs) through the DWT’s use during GSP reviews Cons∙ Timeframe for finalizing DWT did not completely align with timeline for development of GSPs, which limited influence over local groundwater policy design

## Data Availability

Data generated for this manuscript are available from the authors upon request.

## Appendix A

**Table A1:** Drinking Water Tool Layers. This table lists all the reference data layers available in the Drinking Water Tool and provides information about each layer: description, geographic scope, and lead developer. An * indicates that this layer is one of the three interactive layers, that also allows users to view attributes without downloading.

Layer	Description	Geographic Scope	Lead Developer
Groundwater Users			
Community water system service areas boundary *	Polygons for 2851 active community water systems	Statewide	Water Equity Science Shop (WESS) based on Tracking California (2019)
Public supply well location	Point data for 7158 public supply wells with count per location	Statewide	Gailey (2020) based on the online system for well completion reports (OSWCR)
Domestic well points	Point data for 327,252 domestic wells with depth	Statewide	WESS based on OSWCR
Likely domestic well communities	Polygons representing areas likely served by domestic wells, displayed at the 1 × 1 mile grid square	Statewide	WESS
Population reliant on domestic wells	Sum of people per section Sum of housing units per section Count of domestic wells per section	Statewide	WESS
**Water Quality** Arsenic Nitrate Hexavalent chromium 1,2,3-Trichloropropane	Polygons with water quality assigned from the Office of Environmental Health Hazard Assessment’s (OEHHA) CalEnviroScreen 3.0 for community water systems and likely domestic well communities, for each contaminant	Statewide	OEHHA/WESS
**Central Valley Well Impacts** Count of impacted wells Cost to remediate	Total number of domestic wells and municipal wells (for small systems), impacted and cost to remediate impacts from three scenarios of groundwater decline based on a scaled versions of the 2012–2016 drought	San Joaquin Valley	Gailey (2020)
Demographics (Variables are available by three census geographies: place, tract, and block group)			
Median household income (MHI)	MHI values are the estimated five-year averages from the 2017 American Community Survey (ACS) of the US Census	Statewide	GreenInfo Network based on ACS 2013–2017
Disadvantaged communities (DAC)	Geographies with an average MHI of less than 80% of California’s overall MHI (disadvantaged) and less than 60% of the statewide MHI (severely disadvantaged)	Statewide	GreenInfo Network based on ACS 2013–2017
Race	Respondents’ self-identified ethnicity and race from the 2017 ACS five-year average	Statewide	GreenInfo Network based on ACS 2013–2017
Other Boundaries and Jurisdictions			
Counties *	Polygons that represent the 58 counties in California	Statewide	GreenInfo Network based on California Natural Resources Agency (2019)
**Elected Officials:** State Assembly Districts State Senate Districts	80 assembly districts and 40 state senate districts	Statewide	California Redistricting Commission
Groundwater Sustainability Agencies (GSAs) *	Polygons for exclusive GSAs boundaries formed under the Sustainable Groundwater Management Act (SGMA)	Statewide	CWC based on SGMA Data Viewer (Department of Water Resources (DWR) 2019)
Township Boundary	Townships are six-mile square units and indicate the spatial resolution of the water quality data	Statewide	Bureau of Land Management (2019)
Alluvial Boundary	This layer shows the extent of the alluvial deposits in the Central Valley of California and the geographic extent of the Central Valley Well Impacts analyses	Central Valley	United States Geological Survey (2012)
Bulletin 118 Groundwater Basins (B 118 is the official publication on the occurrence and nature of groundwater)			
2019 SGMA Basin Prioritization	This layer shows the SGMA Basin Prioritization of California’s Bulletin 118 groundwater basins and subbasins; visualized by the prioritization levels (very low to high)	Statewide	DWR (2019)
Critically Overdrafted Basins (2018)	This layer shows the 21 groundwater basins that were categorized as critically overdrafted out of California’s Bulletin 118 groundwater basins	Statewide	DWR (2019)

## Appendix B

**Table A2:** Themes from codebook.

Main Code	Sub Code	Code Description
Barriers		Discussions about barriers to using or reasons why the tool was not used, including challenges during the development of the tool.
	Tool adoption/uptake	Discussions about barriers to people using the Drinking Water Tool (DWT).
	Tool development	Discussions about barriers experienced during the DWT development process.
DWT development process		Project-focused, includes discussions of project development process, any discussion of the iterative nature of the DWT development or community based participatory research (CBPR) approach to the DWT.
	Government–academic disconnect	Discussions that get at disconnect or misalignment between researchers/state agencies in terms of priorities, project direction, any territoriality around what data is used, who is an ‘authority’, etc.
	Stakeholder input & feedback	Discussions that are process-based, including feelings and perspectives about what was good/bad/could be done differently.
	Tool ownership	Discussions about who owns the tool
	Transparency	Discussions of transparency (in tool development process, communication), as well as with respect to methods and data transparency.
	Trust and relationships	Discussions of trust building between researchers/non-profits/state agencies, and issues of mistrust.
Environmental justice & Human Right to Water		Discussions of environmental justice/human right to water in relation to the DWT.
Lessons learned & future ideas		Discussions of lessons learned and ideas for the future, such as ways to improve inter-agency collaboration and coordination, ideas for CBPR and efforts in relation to environmental justice and cumulative health-impact research.
Outreach		Discussions about outreach efforts, who the DWT is presented to, quantifying outreach. This applies more to post tool launch activities and is less about tool outreach efforts during tool development.
	Community level/NGO outreach	Discussion of previous or planned community engagement around communicating the DWT’s capabilities, encouraging tool adoption, or building collaborations for future DWT development.
	Interagency coordination and outreach	Discussions of the DWT and data sharing across state agencies and/or research groups, particularly around parallel tools in development.
Tool goal		Statements around the original vision or hope for the DWT.
Tool impact		Statements around an explicit mention of a DWT-related impact.
	Discourse/conversational impact	Discussions of the DWT’s influence on ways in which key components (private well communities, domestic well locations, etc.) are increasingly part of efforts to achieve the goals under the Sustainable Groundwater Management Act (SGMA), the Human Right to Water, and drought and drinking water policy discussions.
	Policy impact	Discussions of indirect, direct, or potential impact of the tool on California water policy, especially around the Human Right to Water, Safe and affordable funding for equity and resilience (SAFER) program, SGMA, and/or drought vulnerability and response.
	Project or research impact	Discussions by the interviewee about ways in which their interaction with the project team or with the tool/data itself is influencing their own project approach, methodological thinking, etc.
	Influence or impact not achieved	Discussions of how or when the DWT did not achieve the expected impact on policy or other arenas (such as policy discourse or projects/research).
